# Mental illness and HIV amongst female inmates in Durban, South Africa

**DOI:** 10.4102/sajpsychiatry.v28i0.1628

**Published:** 2022-01-27

**Authors:** Samantha Naidoo, Ugasvaree Subramaney, Saeeda Paruk, Liezel Ferreira

**Affiliations:** 1 Department of Psychiatry, School of Clinical Medicine, Faculty of Health Sciences, University of the Witwatersrand, Johannesburg, South Africa; 2 Discipline of Psychiatry, Faculty of Health Sciences, University of KwaZulu-Natal, Durban, South Africa

**Keywords:** prevalence, mental illness, female inmates or prisoners, HIV and AIDS, South Africa

## Abstract

**Background:**

There is limited data regarding the prevalence of mental illness and human immunodeficiency virus (HIV) amongst female inmates in South Africa. Rehabilitation programmes can only be formulated once the needs of this population have been identified.

**Aim:**

This study aimed to measure the prevalence of mental illnesses, borderline and antisocial personality disorders and HIV amongst female inmates.

**Setting:**

The study was based at a correctional centre in Durban, KwaZulu-Natal, South Africa.

**Methods:**

This study forms part of a larger two-phased, mixed methods, sequential, explanatory design study. In phase one, 126 female inmates were interviewed using a clinical questionnaire and the Structured Clinical Interview for Diagnostics and Statistical Manual (DSM)-5 diagnoses – Research Version.

**Results:**

The following lifetime prevalence rates were found: depressive disorder 70.6%, alcohol use disorder 48.4%, post-traumatic stress disorder (PTSD) 46.8%, borderline personality disorder 33.3%, substance use disorder 31.7%, antisocial personality disorder 15.1% and psychotic disorder 4.8%. The prevalence of current adult attention-deficit and hyperactivity disorder was 9.5%. A total of 39% of the participants admitted to past suicide attempts, whilst 64.3% reported past suicidal ideation and 36.5% had a current episode of a psychiatric disorder. A total of 64.3% of the participants were living with HIV. Although 90.4% had a lifetime psychiatric disorder, only 16.7% were previously diagnosed with a mental illness. The majority of inmates with lifetime disorders had psychiatric comorbidities.

**Conclusion:**

The high prevalence of mental illness and HIV amongst female inmates, and the fact that most with mental illness remain undiagnosed, is concerning. Improved screening, identification and treatment of mental illnesses in this population is needed to ensure optimal mental health outcomes and decreased recidivism.

## Introduction

There is a paucity of research on the mental health of female prisoners as women constitute a minority of the total prison population, which ranges from 2% to 9% of total prisoners in most countries.^1^ Largely because of the minority status of females in the prison environment, rehabilitation programmes that have been designed for men have been applied to women, without consideration of their gender-specific needs.^2^ These include a higher prevalence of mental disorders such as post-traumatic stress disorder (PTSD), suicide and self-harming behaviours, elevated rates of drug or alcohol dependence, extensive histories of physical and sexual victimisation, medical needs such as reproductive health needs and additional issues related to the women’s responsibility for their children and families.^3^ Furthermore, because of the smaller number of female prisoners, countries often have fewer facilities for women, hence they are often incarcerated far from home.^3^

In the past few decades, there has been a burgeoning interest in female prisoner’s mental health with increased recognition that females have gender-specific vulnerabilities and mental health needs.^4^ Assessment tools and rehabilitation programmes need to take cognisance of this if correctional services are to instil meaningful change in these prisoners. Prisoners, like all other citizens, are entitled to medical treatment which includes mental healthcare. This is a fundamental human right, as enshrined in the South African Constitution (Sections 27 and 35 of the Bill of Rights)^5^ and under international law (Article 25 of the United Nations Universal Declaration of Human Rights).^6^ It has also been observed that prisoners with a co-occurring mental illness and substance use disorder (SUD) have a higher risk of recidivism than inmates with mental illness or SUD alone.^7^ In addition, prisoners with severe mental illnesses (SMIs) such as major depressive disorder (MDD), bipolar disorder, schizophrenia and non-schizophrenic psychotic disorders have a higher risk of recidivism than those without.^8^ Identification and treatment of inmates with mental illnesses, including SUD, should therefore be prioritised by correctional services to decrease recidivism.

Human immunodeficiency virus (HIV) is a global challenge, however, the majority of people living with HIV and/or AIDS (PLWHA) reside in sub-Saharan Africa.^9^ South Africa (SA) has one of the highest prevalence rates of HIV in the world,^10^ and it has the largest number of PLWHA worldwide (7.8 million).^11^ In SA, women are disproportionately affected by HIV with 62.7% of PLWHA being women.^12^ In most countries, the prevalence of HIV amongst prisoners is higher than that of the general population^13^ and in some countries in West and Central Africa, female prisoners have a higher prevalence of HIV than their male counterparts.^13^ Human immunodeficiency virus and mental illness share a complex bi-directional relationship.^14,15^ Thus, it is important to determine the burden of HIV amongst female inmates in KwaZulu-Natal (KZN), the province with the highest prevalence of HIV in SA.^16^

### Prison populations

In 2018, worldwide, there were an estimated 10.74 million prisoners (including remand detainees and sentenced prisoners).^17^ A total of 70% of the world’s prisoners come from low- and middle-income countries (LMICs).^18^ In 2017, the female prison population worldwide was approximately 714 000, which is approximately 6.9% of the total prison population.^1^ The total female prison population has grown by approximately 53.3% since the year 2000.^1^ South Africa is ranked 12th highest amongst the world’s total prison population and 45th in terms of prisoner rate per 100,000 of the population.^19^ Currently there are a myriad of challenges facing correctional centres in SA, as is the case in most LMICs, which include inter alia, overcrowding, human rights violations such as torture and assault, the scourge of infectious diseases such as tuberculosis (TB), HIV and acquired immunodeficiency syndrome (AIDS),^20^ ailing prison infrastructure, inadequate staffing with a resultant inability to offer adequate rehabilitation services, illicit drug addiction, and also gangsterism and corruption.^21^

### Mental illnesses amongst prisoners

An updated systematic review on 33 588 prisoners from 24 countries worldwide from 1966 to 2010, found little change in the prevalence of SMIs amongst prisoners in the past decade, with psychotic disorders amongst female prisoners averaging 3.9% (3.6% for males) and depressive disorders averaging 14.1% in females (10.2% in males).^22^

Another common mental illness amongst prisoners is PTSD.^23^ A 2007 systematic review of sentenced prisoners reported prevalence rates of PTSD ranging from 4% to 21%, with females being disproportionately more affected than males.^23^ A more recent systematic review found the pooled point-prevalence of PTSD in female prison populations to be 21.1%.^24^

Substance use disorders and alcohol use disorders (AUDs) are also amongst the most common disorders diagnosed in prisoners, with females having higher rates of addiction than males for illicit substances but lower rates for alcohol.^25^ Commonly abused substances include alcohol, cannabis, stimulants and opiates.^25,26,27^

Most children and adolescents with attention deficit and hyperactivity disorder (ADHD) will continue to suffer from the disorder in adulthood,^28^ having worse outcomes on academic, career, health, social and even personal safety if untreated.^29^ There is a disproportionately higher prevalence of ADHD in individuals in the criminal justice system than in the general population.^30^ A recent meta-analysis of 42 studies conducted in 15 countries found a prevalence of 25.5% of ADHD amongst incarcerated populations with no significant difference between males and females.^30^ ADHD is also associated with early age criminality and an increased rate of recidivism.^31^

Incarcerated populations also have an over-representation of personality disorders.^32^ In the initial systematic review by Fazel and Danesh in 2002, 21% of female prisoners had antisocial personality disorder (ASPD) and 25% had borderline personality disorder (BPD).^32^ There is a strong association between prisoners and ASPD. However, the prevalence of ASPD amongst female prisoners is generally lower than in males.^33,34^ There is a preponderance of BPD amongst female prisoners.^32,33,34,35^

There are limited studies on prisoners in LMICs, particularly in Africa. A systematic review on the prevalence of SMIs amongst prisoners in LMICs revealed a 1-year prevalence rate of 6.2% for psychosis, 16.0% for depression, 3.8% for AUDs and 5.1% for SUDs.^18^ To date, there has been only one study on the prevalence of mental disorders amongst offenders in KZN, SA.^36^ The major limitation of that study was the significant male gender bias with only 3 of the 193 participants interviewed being females. The study found that 55.4% of offenders had a DSM-4 Axis 1 psychiatric disorder, the most common being AUDs and SUDs (42%). A total of 23% of the participants were diagnosed with current psychotic, bipolar, depressive and anxiety disorders and 46.1% had an ASPD. Notably, most offenders who had mental disorders were neither detected nor treated by correctional mental health services at the time.^36^ The above-mentioned studies illustrate the hiatus in the literature with respect to the prevalence of mental illnesses amongst female inmates in SA, therefore in this study, we aim to describe the prevalence of mental illnesses and HIV in female inmates in a South African correctional centre.

### Definitions

1. Inmate: ‘Any person, whether convicted or not, who is being detained in custody in any correctional centre or remand detention facility’.^37^ ‘Inmate’ is used in this study as per the *Correctional Services Act* (CSA) 111 of 1998 of SA, however, the word ‘prisoner’ is used more commonly in international research.^1,2,3,4,6,7,8,13,17,18,19,23,24,25,26,27,30,31,32,33,34,35^
2. Remand detainee (RD): ‘A person detained in a remand detention facility awaiting the finalisation of his or her trial, whether by acquittal or sentence, if such person has not commenced serving a sentence or is not already serving a prior sentence’.^37^
3. Sentenced offender (SO): ‘A convicted person sentenced to incarceration or correctional supervision’.^37^
4. Correctional facility: ‘Any place established under the Correctional Services Act 111 of 1998 of South Africa as a place for the reception, detention, confinement, training or treatment of persons liable to detention in custody or to placement under protective custody’.^37^ ‘Correctional facility’ is used in this study as per the CSA 111 of 1998, however, the commonly used term internationally is ‘prison’.
5. Recidivism: The tendency of a convicted criminal to re-offend.^38^ Recidivism was calculated as the number of participants (SOs and RDs) who had previously been convicted of an offence.
6. Violent offences: Offences where a person is harmed by or threatened with violence, which for the purposes of this study include murder, attempted murder, robbery, robbery with aggravating circumstances, assault, assault with intent to do grievous bodily harm (GBH), rape, conspiracy to murder, human trafficking and kidnapping.^39,40^
7. Non-violent offences: Offences in which no injury or physical force or threat of force is used against a person, which for the purposes of this study include theft, fraud, housebreaking, racketeering, corruption, money laundering, dealing in drugs, possession of drugs, forgery and uttering, contravening the Medicines and Related Substances Act, breaking parole, possession of stolen property and unlawful possession of a firearm.^39,40^

## Method

### Study design

This study formed part of a larger two-phased, mixed methods, sequential, explanatory design study,^41^ which examined the mental health needs of female inmates in Durban, SA. Phase one was quantitative, cross-sectional and descriptive whilst phase two was qualitative. The findings reported here are part of the first phase, which aimed to describe the prevalence of mental illness, HIV and trauma in this population. This manuscript reports on the prevalence of mental illnesses and HIV in this population.

### Study setting

This correctional centre, situated in Durban, KZN is one of the largest correctional centres in sub-Saharan Africa. However, it remains a largely under-researched area geographically. It accommodates male and female inmates. At the time of the study, the female section of this correctional facility had two full-time social workers and two part-time psychologists who consulted on an ad hoc basis. In addition, there was one psychiatrist consulting part-time for the entire correctional facility.

### Study sample

At the time of the study there were 349 female inmates at this correctional centre, consisting of 250 SOs and 99 RDs. Inclusion criteria for phase one of the study included all adult female inmates (18 years and older), who were able to provide written informed consent. Capacity to provide consent was assessed by the first author. The RDs and SOs who spoke either English or isiZulu were included. Exclusion criteria were participants who lacked capacity to provide informed consent, including those who were floridly psychotic and behaviourally disturbed such that they might pose a danger to themselves or others. For women who were illiterate, the contents of the information leaflet were explained and informed consent was obtained via thumbprint. Stratified random sampling for SOs and RDs was conducted. A total of 126 women participated in phase one interviews.

### Instruments

#### Socio-demographic, clinical and forensic questionnaire

A socio-demographic, clinical and forensic questionnaire, based on a review of the literature was compiled and administered by the first author. The forensic component of this interview elicited information regarding details of the current and past charges and/or convictions, circumstances surrounding the offence, motivation for the offence and details of referral for forensic observation (if applicable). Information regarding offence and sentence was confirmed from each participant’s official prison cards. The clinical component contained questions about current and past medical illnesses (including HIV), past psychiatric illnesses (including self-harming behaviours, suicidal ideation and attempts) and relevant treatment thereof.

#### Structured clinical interview for Diagnostics and Statistics Manual of mental disorders 5th edition – Research Version

The SCID-5 is a structured clinical interview that can be used to assess mental disorders and provide diagnoses according to the definitions and criteria of the American Psychiatric Association’s Diagnostics and Statistical Manual. The Research Version, non-patient edition of the SCID was administered in this study.^42^ Only the modules diagnosing psychotic, depressive, bipolar, PTSD, AUDs, SUDs, adult ADHD, ASPD and BPD were administered. The SCID measures both current and lifetime prevalence of psychiatric disorders. It also describes stressors precipitating PTSD. The Structured Clinical Interview for Diagnostics and Statistical Manual (DSM)-5 Personality Disorders (SCID 5-PD) was used for the diagnosis of ASPD and BPD. Although the current version of the SCID has not been standardised for the South African population, the DSM-5 remains the gold standard for diagnosing mental illness.

### Procedure

Data collection was from August to November 2019. The study assessments were conducted by the first author who is a forensic psychiatrist. Prior to commencement of the study, all 349 adult female inmates were addressed and introduced to the study. They were informed that their participation would be voluntary, anonymous and confidential. In addition, they were informed that the first author was not affiliated to the Department of Correctional Services (DCS) and that their participation in the study would in no way influence their criminal proceedings. They were then invited to participate in the study. Inmates who were not keen to participate after being addressed were not included in the sampling frame. Stratified random sampling of both groups of inmates (SOs and RDs) was conducted. Most interviews were conducted in English, however, for a minority of women who spoke isiZulu only, an English–isiZulu translator assisted. The translator was a qualified social worker with a research background and was bilingual.

### Data collation

Data was captured online by the first author on-site, using Research electronic data capture (Redcap). Redcap is a browser-based, meta-data driven electronic data capture software solution and workflow methodology for designing clinical and translational research data bases.^43^

### Analysis

Data from both the SCID-RV (regarding diagnosis) and the clinical questionnaire (regarding other clinical information) were analysed using IBM Statistical Package for the Social Sciences (SPSS) version 26. Frequency tables with percentages and graphs were used to describe categorical variables. Quantitative variables were summarised using mean and standard deviation (s.d.). Categorical variables were compared between groups using chi-square tests or Fisher’s exact tests as appropriate. A *p* < 0.05 indicated statistical significance.

### Ethical considerations

Approval for this study was obtained from the University of the Witwatersrand Human Research Ethics Committee (reference number: M181026), and from the DCS. Written informed consent was obtained from all participants. Participants’ HIV status were confirmed with their informed consent, using prison hospital records. Those who had a mental illness/psychological distress, which required urgent intervention were referred, with their consent, to the prison doctor or psychologist. Data were stored electronically and were password protected. The DCS had no access to any information derived from the participants. Participants were advised on their rights not to participate, voluntariness of participation and their right to withdraw at any time without impacting their care at the correctional facility. Undue influence was minimised as the first author was independent of the DCS and offered no financial incentive to participate. Women who participated were given a hygiene pack with sanitary items valued at R60.

## Results

Table 1 shows the socio-demographic profile of the 126 female inmates (96 SOs and 30 RDs) who participated.

**TABLE 1:** Socio-demographic profile of 126 participants (SOs and RDs).

Demographics	*N*	%
**Home language**		
English	18	14.3
Zulu	97	77.0
Afrikaans	2	1.6
Xhosa	6	4.8
Sotho	3	2.4
**Nationality**		
South African	125	99.2
Other	1	0.8
**Residential area**		
Rural	31	24.6
Urban	95	75.4
**Population group (self-reported)**		
Black people	106	84.1
White people	4	3.2
Mixed race	4	3.2
Indian	12	9.5
**Highest level of education**		
No formal schooling	3	2.4
Less than primary school	14	11.1
Primary school completed	57	45.2
Secondary/High school completed	31	24.6
College/University completed	18	14.3
Postgraduate degree	3	2.4
**Employment status prior to incarceration**		
Government employee	15	11.9
Non-government employee	40	31.7
Self-employed	21	16.7
Student	4	3.2
Unemployed (able to work)	44	34.9
Unemployed (unable to work)	2	1.6
**Civil status prior to incarceration**		
Married	35	27.8
Living as couple	17	13.5
Divorced or separated	8	6.3
Single	56	44.4
Widowed	10	7.9
**Religion**		
Christianity	120	95.2
Islam	3	2.4
Hinduism	2	1.6
Other	1	0.8
**Total personal monthly income prior to incarceration**		
Less than R1000	47	37.3
R1000–R2000	19	15.1
R2001–R5000	25	19.8
R5001–R10 000	9	7.1
More than R10 000	26	20.6

### Forensic profile

**FIGURE 1:**
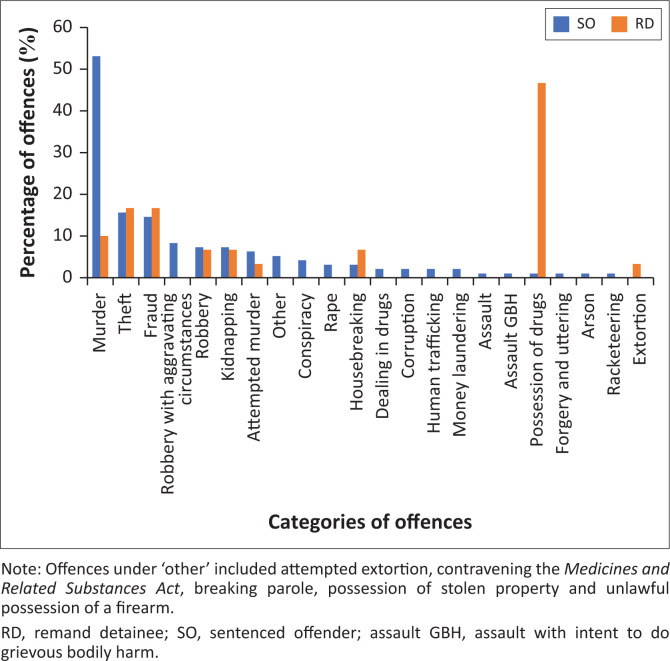
Categories of current offences.

The mean age at first arrest was 32 years (s.d. = 10.01). A total of 32% (*n* = 41) of the participants were recidivists. Most women (73.8%, *n* = 93) were incarcerated for a single offence, however, more than a quarter (26.2%, *n* = 33) were incarcerated for two or more offences. A total of 3% (*n* = 4) of the participants had been referred for forensic observation whilst awaiting trial. Three were found fit to stand trial and criminally responsible (and subsequently found guilty and sentenced) whilst one was currently awaiting trial. This number does not reflect those who were found not fit or not responsible, and who were diverted to forensic psychiatric hospitals for care, treatment and rehabilitation under the *Mental H ealth Care Act* 17 of 2002.^44^ Figure 1 shows the categories of current offences.

### Clinical profile

Table 2 shows the prevalence of DSM-5 disorders. Psychotic disorders (past month) consisted of 0.8% (*n* = 1) schizophrenia and 0.8% (*n* = 1) unspecified psychotic disorder. Psychotic disorders (lifetime) consisted of 1.6% (*n* = 2) psychotic disorders due to another medical condition, and 1.6% (*n* = 2) unspecified psychotic disorders, and those listed under psychotic disorders (current). Depressive disorders (past month and lifetime) were all in the category of MDD.

**TABLE 2:** Prevalence of DSM-5 disorders.

Variables	RD (*n* = 30)		SO (*n* = 96)		Total (*n* = 126)	
	*n*	%	*n*	%	*n*	%
Psychotic disorders (past month/current)	1	3.3	1	1.0	2	1.6
Psychotic disorders (lifetime)	1	3.3	4	4.2	6	4.8
Depressive disorders (past month/current)	5	16.7	7	7.3	12	9.5
Depressive disorders (lifetime)	19	63.3	70	72.9	89	70.6
Bipolar disorders (past month/current)	0	0.0	0	0.0	0	0.0
Bipolar disorders (lifetime)	1	3.3	0	0.0	1	0.8
PTSD (past month/current)	1	3.3	0	0.0	1	0.8
PTSD (lifetime)	16	53.3	43	44.8	59	46.8
ADHD (past six months/current)	3	10.0	9	9.4	12	9.5
Borderline personality disorder	9	30.0	33	34.4	42	33.3
Antisocial personality disorder	2	6.7	17	17.7	19	15.1

RD, remand detainee; SO, sentenced offender; PTSD, post-traumatic stress disorder; ADHD, attention deficit and hyperactivity disorder.

A minority of women with PTSD had experienced more than one episode during their lifetime. A total of 38 out of the 59 women (64.4%) reported rape as having been the precipitating stressor. Two women (3.4%) had experienced PTSD after being hijacked. Ten women (16.9%) had witnessed stabbings, shootings, suicide or murder. Five (8.5) had been stabbed, whilst one (1.7%) had been shot in the head. Five (8.5%) had been robbed at either knifepoint or gunpoint, whilst three women (5.1%) had been involved in a motor vehicle accident.

#### Alcohol and substance use disorders

**FIGURE 2:**
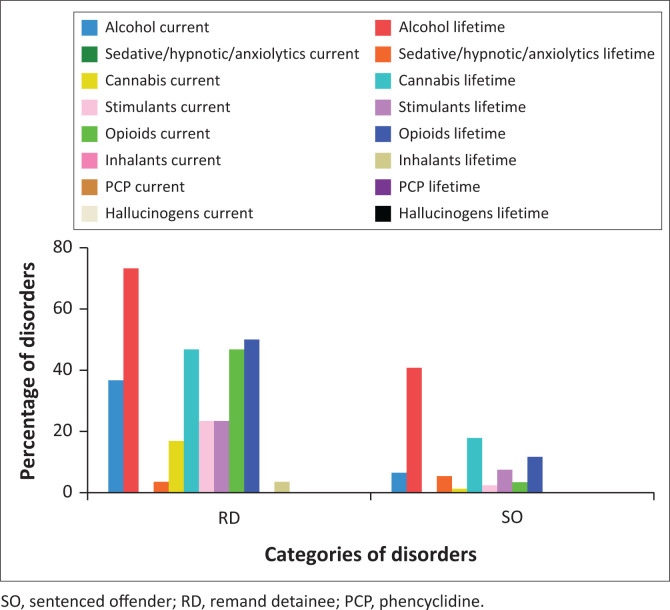
Prevalence of alcohol use disorders and substance use disorders among remand detainees and sentenced offenders.

Figure 2 shows the prevalence of AUDs and SUDs. We found 13.5% of women had a 12-month prevalence of an AUD, whilst 48.4% (*n* = 61) had a lifetime prevalence of an AUD. Overall 18.3% (*n* = 23) had a 12-month prevalence of a SUD, whilst 31.7% (*n* = 40) had a lifetime prevalence of a SUD.

#### Self-harming behaviour and suicidality

The lifetime prevalence of non-fatal deliberate self-harming behaviours, which included cutting and burning was 5.6% (*n* = 7). The lifetime prevalence of suicide attempts was 39.7% (*n* = 50) and the most common method of attempting suicide was overdosing on medication. Many women (64.3%, *n* = 81) admitted to past suicidal ideation.

#### Psychiatric comorbidities

A total of 36% of the women (*n* = 46) had a current episode of a psychiatric disorder (i.e. psychotic, depressive, PTSD, AUDs or SUDs) or a current psychiatric disorder (adult ADHD) and 7.9% (*n* = 10) experienced two or more psychiatric disorders currently, whilst 90.4% (*n* = 114) had a lifetime diagnosis of a psychiatric disorder with 62.7% (*n* = 79) having two or more lifetime diagnoses.

#### Past psychiatric history

A total of 16% (*n* = 21) had a prior psychiatric diagnosis; however, most women (90.4%) were found to have suffered from a lifetime psychiatric disorder.

#### HIV and mental illness

The prevalence of HIV was 64.3% (*n* = 81) with all, except one participant, being on highly active anti-retroviral therapy currently. Table 3 shows the association between HIV and mental illness. There was a statistically significant association between HIV and PTSD and HIV and AUD. Those participants with PTSD were more likely to be HIV positive than those without PTSD (79.7% vs. 50.7%, *p* = 0.001). The participants with AUD were also more likely to be HIV positive than those without AUD (75.4% vs. 53.8%, *p* = 0.012). There was a non-statistically significant trend shown in those with a SUD or AUD (lifetime) and HIV (71.6% vs. 55.9%; *p* = 0.066). Other non-significant trends observed to be more common amongst PLWHA were depressive disorders, SUDs and psychotic disorders. However, a larger study is required to explore these findings further.

**TABLE 3:** Associations between HIV and mental illnesses.

Variables	Offences						*p*
	HIV negative		HIV positive		Total		
	*n*	%	*n*	%	*n*	%	
**Depressive disorders (lifetime)**							0.466
No	15	40.5	22	59.5	37	100.0	
Yes	30	33.7	59	66.3	89	100.0	
Total	45	35.7	81	64.3	126	100.0	
**Bipolar disorders (lifetime)**							0.357
No	44	35.2	81	64.8	125	100.0	
Yes	1	100.0	0	0.0	1	100.0	
Total	45	35.7	81	64.3	126	100.0	
**SUD or AUD (lifetime)**							0.066
No	26	44.1	33	55.9	59	100.0	
Yes	19	28.4	48	71.6	67	100.0	
Total	45	35.7	81	64.3	126	100.0	
**SUD (lifetime)**							0.189
No	34	39.5	52	60.5	86	100.0	
Yes	11	27.5	29	72.5	40	100.0	
Total	45	35.7	81	64.3	126	100.0	
**PTSD (lifetime)**							0.001
No	33	49.3	34	50.7	67	100.0	
Yes	12	20.3	47	79.7	59	100.0	
Total	45	35.7	81	64.3	126	100.0	
**Psychotic disorders (lifetime)**							0.420
No	44	36.7	76	63.3	120	100.0	
Yes	1	16.7	5	83.3	6	100.0	
Total	45	35.7	81	64.3	126	100.0	
**ADHD (current)**							0.346
No	39	34.2	75	65.8	114	100.0	
Yes	6	50.0	6	50.0	12	100.0	
Total	45	35.7	81	64.3	126	100.0	
**AUD (lifetime)**							0.012
No	30	46.2	35	53.8	65	100.0	
Yes	15	24.6	46	75.4	61	100.0	
Total	45	35.7	81	64.3	126	100.0	

HIV, human immunodeficiency virus; AUD, alcohol use disorders; SUD, substance use disorder; PTSD, post-traumatic stress disorder.

#### Mental illness and offending

Table 4 shows the association between offending and mental illness and offending and personality disorders. Having an AUD or SUD was significantly associated with non-violent offences (*p* = 0.001). A total of 52% participants with SUD or AUD committed non-violent offences whereas 23.7% of those without SUD or AUD committed non-violent offences. Similarly, SUD and AUD were independently associated with non-violent offences (*p* = 0.002 and 0.002, respectively). The most common non-violent offence was drug possession. Conversely, violent offences were associated with not having an AUD or SUD. The most common violent offence was murder. Borderline personality disorder was non-significantly higher in women who committed violent offences (*p* = 0.093).

**TABLE 4:** Associations between offending and mental illnesses and personality disorders.

Variables	Offences						*p*
	Non-violent		Violent		Total		
	*n*	%	*n*	%	*n*	%	
**Depressive disorders (lifetime)**							0.164
No	18	48.6	19	51.4	37	100.0	
Yes	31	34.8	58	65.2	89	100.0	
Total	49	38.9	77	61.1	126	100.0	
**Bipolar disorders (lifetime)**							0.389
No	48	38.4	77	61.6	125	100.0	
Yes	1	100.0	0	0.0	1	100.0	
Total	49	38.9	77	61.1	126	100.0	
**AUD or SUD (lifetime)**							0.001
No	14	23.7	45	76.3	59	100.0	
Yes	35	52.2	32	47.8	67	100.0	
Total	49	38.9	77	61.1	126	100.0	
**SUD (lifetime)**							0.002
No	25	29.1	61	70.9	86	100.0	
Yes	24	60.0	16	40.0	40	100.0	
Total	49	38.9	77	61.1	126	100.0	
**PTSD (lifetime)**							0.984
No	26	38.8	41	61.2	67	100.0	
Yes	23	39.0	36	61.0	59	100.0	
Total	49	38.9	77	61.1	126	100.0	
**Psychotic disorders (lifetime)**							0.667
No	46	38.3	74	61.7	120	100.0	
Yes	3	50.0	3	50.0	6	100.0	
Total	49	38.9	77	61.1	126	100.0	
**ADHD (current)**							0.213
No	42	36.8	72	63.2	114	100.0	
Yes	7	58.3	5	41.7	12	100.0	
Total	49	38.9	77	61.1	126	100.0	
**AUD (lifetime)**							0.002
No	17	26.2	48	73.8	65	100.0	
Yes	32	52.5	29	47.5	61	100.0	
Total	49	38.9	77	61.1	126	100.0	
**Borderline personality disorder**							0.093
No	37	44.0	47	56.0	84	100.0	
Yes	12	28.6	30	71.4	42	100.0	
Total	49	38.9	77	61.1	126	100.0	
**Antisocial personality disorder**							0.843
No	42	39.3	65	60.7	107	100.0	
Yes	7	36.8	12	63.2	19	100.0	
Total	49	38.9	77	61.1	126	100.0	

AUD, alcohol use disorders; SUD, substance use disorder; PTSD, post-traumatic stress disorder.

## Discussion

The main findings of this study were the high prevalence of mental illnesses and HIV amongst female inmates compared with the general population in SA.^45^ This elevated prevalence of major psychiatric disorders amongst prisoners compared with the general population is also in keeping with prevalence rates in other LMICs settings.^18^

The estimated overall HIV prevalence rate in SA is approximately 13%. In the age group 15–49 years, an estimated 18.7% of the population is living with HIV.^11^ Studies have consistently shown a higher prevalence of HIV amongst women than men in SA particularly in KZN province,^46^ with women in some parts of KZN having an HIV prevalence of as high as 60%.^47^ Cultural practices, the low socio-economic status of women and gender-based violence are some of the reasons cited to explain this disparity.^48^ A review of HIV amongst prisoners in sub-Saharan Africa found prevalence rates ranging from 2.3% to 34.9%, which were almost always higher than that of the non-incarcerated population in the same country.^49^ Although our findings are in keeping with the literature from sub-Saharan Africa,^49^ the extremely high number of women in our study living with HIV, and the association of HIV with mental illnesses, are concerning and should be a focus of targeted interventions at this correctional facility.

The South African Stress and Health study (SASH), which was the first large population-based mental health epidemiological survey in South Africa, measured the 12-month and lifetime prevalence of mental illness amongst the general population in all 9 provinces.^45^ However, psychotic disorders, bipolar disorders, adult ADHD and personality disorders were not measured in the SASH study.

In Fazel and Seewald’s updated review 3.9% had a 6-month prevalence of psychotic disorders, which is higher than in our study (1.6% one-month prevalence).^22^ The 1-year pooled prevalence for psychosis in the systematic review in LMICs was 6.2%.^18^ Possible reasons to account for the lower rate in our sample may be that we measured 1-month prevalence compared with the other studies, which measured 6-month and 1-year prevalence. In addition, it has been reported that prevalence of psychotic disorders is higher on admission to prison, with decreased rates found in inmates with longer time spent in prison.^18^ The majority of our sample were SOs, which may account for the lower prevalence of psychosis. Furthermore, accused who were referred for forensic observation and found not fit or not responsible on the basis of a psychotic disorder were diverted to forensic psychiatric hospitals instead of the criminal justice system.^44^

Fazel and Seewald’s updated review also found a 6-month prevalence of 14.1% for depression,^22^ whilst the 1-year pooled prevalence for MDD in the systematic review in LMICs, by Baranyi et al., was 16.0%^18^, which are both higher than in our study (9.5%). Possible reasons for the lower prevalence of current MDD in this study may again be accounted for by the shorter 1-month prevalence we measured as compared with the other studies and that the majority of participants in our study were SOs. Higher rates of depression have been reported in prisoners on admission as was found in the systematic review in LMICs.^18^ This was also borne out in our study where RDs had more than double the rate of current depression compared with SOs. This may be because of adjustment to prison posing a huge stressor for women. The lifetime prevalence of depressive disorders in our study (70.6%) is substantially higher than in the SASH study, which found that 4.9% of the general population had a 12-month prevalence of MDD, whilst 9.8% had a lifetime prevalence.^45^ Thus, the inmate population carries a far heavier burden of depressive disorders than the general population. A study from Ethiopia suggests that possible risk factors for this high prevalence of depression amongst inmates includes a family history of mental illness, poor social support, comorbid medical disorders, concern about life after release from prison, substance use and having a history of previous incarceration.^50^

The lifetime prevalence of PTSD in this study (46.8%) is much higher than in the SASH study, which found a 2.3% lifetime prevalence in the general population^45^ but is consistent with a meta-analysis, which found a lifetime prevalence of 40.4% in female prison populations.^24^ There was a high level of traumatic experiences including rape, stabbings and shootings in our sample of women, which is in keeping with the high prevalence of gender-based violence in SA.^51,52^ Both physical assault and rape are traumas most likely associated with PTSD.^53,54^ In addition, our study found a significant association between PTSD and PLWHA. Post-traumatic stress disorder may either precede an HIV diagnosis because of previously experienced traumatic events, for example, rape or sexual assault or PTSD may appear during the course of the HIV illness because of traumatic incidents experienced.^55^ Despite considerable variation in the rates of PTSD amongst PLWHA, the literature suggests that their rate of PTSD is high.^56,57,58,59^ A meta-analysis in 2012 demonstrated highly disproportionate rates of trauma exposure and recent PTSD in HIV infected women compared with the general population.^60^ People living with HIV and/or AIDS and PTSD are less likely to adhere to their medication regimens and are less likely to practise safe sex with their partners.^61^

A meta-analysis of ADHD amongst young adults in the general population found a prevalence of 5.0%,^62^ whilst the meta-analysis amongst prisoners by Young et al. revealed a prevalence of 25.5% with adult females having a non-significantly lower prevalence of 22.1%.^30^ In this meta-analysis on prisoners, European countries had the highest prevalence of ADHD, followed by North America, however, there was significant heterogeneity. Lower rates of ADHD amongst prisoners have been reported in two recent high-quality studies, conducted on Canadian and French male prisoners, using self-report measures and diagnostic instruments. They reported ADHD prevalence rates of 16.5% and 11%, respectively.^63,64^ Tyler et al. in their study across 13 prisons in the United Kingdom (UK) found that 7.3% of female prisoners screened positive for ADHD, which is similar to the prevalence in our study.^65^ The prevalence of adult ADHD in our study (9.5%) is similar to that of the French and British studies but lower than that of the systematic review. Possible reasons for this are that our study measured the current prevalence of ADHD only and that different instruments were used in the other studies.

The prevalence of AUD was higher in this study than in the general population in the SASH study, which was 5.7% (12 month prevalence) and 14% (lifetime prevalence).^45^ In the SASH study, 1.5% of the general population had a 12-month prevalence of a SUD, whilst 4.5% had a lifetime SUD.^45^ In another systematic review of prisoners, Fazel et al. found a 12-month pooled prevalence of 20% for AUDs and 51% for SUDs in females,^25^ thus our sample had a lower rate of AUDs and SUDs compared with international prison populations, but higher rates compared with the South African general population. In addition, our study found an association between AUD and PLWHA. A 2019 systematic review found that PLWHA and AUD are at greater risk of poor medication adherence, unsafe sex practices and poor quality of life.^66^

A total of 33% of the women in our study were diagnosed with BPD, which is higher than that found in Fazel and Danesh’s systematic review (25%).^32^ Furthermore, BPD was found to be non-significantly higher in those who had been charged with violent offences. This may be an area for future research as existing literature shows associations between BPD and violent offending.^67^ In addition, the high prevalence of past suicide attempts and suicidal ideation is alarming and should be an area of treatment focus because South Africa’s suicide rate (11.15 per 100 000) is more than the global average of 10 per 100 000.^68^ Suicide is the leading cause of death in custody globally and is preventable; hence, it needs to be targeted in this high-risk population.^69^

In our study, a minority of women (16.7%) had a previous psychiatric diagnosis and/or treatment for a psychiatric disorder despite the majority having a lifetime history of mental illness. This is in contrast to the study by Tyler et al. (UK) who found that a high number of prisoners had previous contact with mental health services or a prior psychiatric diagnosis.^65^ South Africa, like most other LMICs in sub-Saharan Africa, has a significant treatment gap with respect to mental healthcare resources available to identify and treat the large burden of mental illnesses in the general population. It is estimated that this treatment gap is approximately 80% in KZN.^70^ There is currently no data describing the treatment gap for prison services in SA. Although the treatment gap was not directly measured in the quantitative phase of this study, the treatment gap may be inferred from the number of women diagnosed with a lifetime mental illness (90.4%) versus the number of women diagnosed with, and/or treated for a mental illness previously (16.7%). This significant gap is likely to exist in other correctional centres in SA and will need to be addressed if correctional services are to achieve optimal mental health outcomes and decreased recidivism.

### Limitations

This study was based at one correctional centre in KZN. Another limitation was that the study relied on retrospective self-report and this information was vulnerable to exaggeration, misinterpretation and distortions, particularly when diagnosing lifetime disorders. Neuro-cognitive disorders, which are common in prison populations and in PLWHA, were not measured in this study. A further limitation was the lack of collateral information, which would have enhanced diagnostic accuracy. As a result of the cross-sectional design of the study, we were unable to draw causal inferences about associations found. Prisoners assessed by lay interviewers have been associated with higher prevalence rates of mental illness than interviews conducted by clinically trained psychologists and psychiatrists. The strengths of this study were that all interviews were conducted by a forensic psychiatrist. In addition, the SCID 5-RV, which is the gold standard for diagnosing mental illness, was used.

## Conclusion

This is the first study which measured the prevalence of mental illnesses and HIV amongst female inmates at a correctional centre in South Africa. Although conducted at one site, this study demonstrates the substantial burden of mental illness and HIV prevalent amongst female inmates compared with international female prison populations as well as to the general population in SA. This underscores the critical need to improve mental health screening, treatment, care and rehabilitation services in correctional centres in SA. Screening should ideally be conducted at admission so that individualised programmes may be tailored and implemented during incarceration. Surveillance and monitoring should be ongoing. Incarceration serves as an opportunity for the implementation of integrated mental health programmes including substance rehabilitation and trauma-focused interventions. Robust interventions during incarceration may positively impact women’s future behaviour and decrease their rate of recidivism. It is recommended that further research be undertaken at all correctional centres in SA to facilitate comparison and more importantly to form a knowledge base for the formulation and implementation of mental health care programmes designed specifically for female inmates.
